# Exploring MAPT-containing H1 and H2 haplotypes in Parkinson’s disease across diverse populations

**DOI:** 10.1038/s41531-026-01394-9

**Published:** 2026-06-24

**Authors:** Paula Reyes-Pérez, Jia Wei Hor, Tzi Shin Toh, Arinola O. Sanyaolu, Caroline B. Pantazis, Thiago Peixoto Leal, Sheila Yeboah, Sara Bandres-Ciga, Huw R. Morris, Mary B. Makarious, Konstantin Senkevich, the Latin American Research Consortium on the Genetics of Parkinson’s Disease (LARGE-PD), Pedro Chana-Cuevas, Ximena Pizarro Correa, Angel Vinuela, Esther Colon, Jorge Orozco, Beatriz Munoz Ospina, Griselda Alvarado, Luciana Rojas-Vazquez, Juan Pablo Diaz-Rearte, Cesar Avila, Egberto Reis Barbosa, Artur F. S. Schuh, Carlos R. M. Rieder, Gabriela Magalhães Pereira, Deise Cristine Friedrich, Thalya Osmilda Alves de Carvalho, Isabella Fonseca Benati, Jullivan Käfer Pasin, Vitor Picanço Lima Gomes, Marcelo Somma Tessari, Ingrid Lorena da Silva Gomes, Juan Sebastián Sánchez León, Paula Saffie-Awad, Gabriel Alves Marconi, Manoella Guatimuzim Testa da Silva, Eduardo Drews Amorim, Vitor Tumas, Angela Vieira Pimentel, Gabriel R. Vilela, Joyce Yamamoto, Carolina P. Souza, Ana L. N. Cunha, Reyna M. Durón, Alex Medina, Heike Hesse, Evelin Alvarez Herrera, Eduardo P. Murillo, Glenda Oliva, Valentina Muller, Matías López Razquin, Lucia Wang, Clarisa Marchetti, Bruno Lopes Santos-Lobato, Gracivane Lopes Eufraseo, Juliana dos Santos Duarte, Marcella Montenegro, Tatiane Souza, Camille Sena, Ândrea Ribeiro-dos-Santos, Grace Letro, Sergio Rodriguez Quiroga, Emilia Gatto, Claudia Perandones, Martin Radrizani, Gustavo DaPrat, Natalia Gonzalez Rojas, Melisa Espindola, Martin Cesarini, Pedro Braga-Neto, Deborah Rangel, Marconny Cavalcante, Mateus Balsells, Julia Rios Pinto, Angel Medina, Ivan Cornejo-Herrera, Koni Mejia, Mario Cornejo-Olivas, Mayela Rodríguez-Violante, Amin Cervantes-Arriaga, Daniel Martinez Ramirez, David Aguillon, Sonia Moreno, Omar Buritica, David Pineda, Marlene Jimenez-Del-Rio, Carlos Vélez-Pardo, Sarita Firstman, Patricio Olguín, Alicia Colombo, Juan Cristobal Nuñez, Andres De la Cerda, María Francisca Canals, Gonzalo Farías, Valentina Bessa, Mérida Terán, Gabriel Torrealba-Acosta, Tania Lobo-Prada, Jaime Fornaguera-Trías, Álvaro Hernandez-Guillen, Susana Lissette Peña Martínez, Tatiana Ascencio, Oscar Peña Rodas, Sarael Alcauter, Alejandra Medina-Rivera, Alejandra E. Ruiz-Contreras, Miguel Rentería, Alejandra Lázaro-Figueroa, Juan Manuel Esquivias-Farias, Andrés Morales-de-Arcia, Alejandra Zayas-Del Moral, Damaris Vazquez-Guevara, Yamil Matuk-Pérez, Carlos Manuel Guerra-Galicia, Ildefonso Rodriguez-Leyva, Karla Salinas-Barboza, Eugenia Morelos-Figaredo, Omar Cardenas-Saenz, Nadia A. Gandarilla-Martínez, Sara Isais-Millán, Teresa Pérez-Torres, Domingo Martinez, Ingrid Estada-Bellmann, Roberto Trejo-Ayala, Carlos Alberto Ponce-Fernández, Dante Oropeza, Oscar Bernal Pacheco, Tatiana Lopez Gonzalez, Humberto Arboleda, Carlos Eduardo Arboleda Bustos, Hebert Bernal Castro, Juan David Caicedo Narvaez, Kelly Bonilla Vargas, Elena Dieguez, Vanderci Borges, Carolina Candeias da Silva, Henrique Ballali Ferraz, Dayany Leonel Boone, Mariana Cavalcanti, Karen Nuytemans, Anisley Martinez, Liena Infante, the Global Parkinson’s Genetics Program (GP2), Jorge Orozco, Karen Nuytemans, Jifeng Guo, Piu Chan, Wei Luo, Zhenhua Liu, Gonzalo Arboleda, Marlene Jimenez del Rio, Alvaro Hernandez, Per Borghammer, Mohamed Salama, Walaa A. Kamel, Yared Z. Zewde, Alexis Brice, Jean-Christophe Corvol, Mari Vidailhet, Mariam Kekenadze, Irine Khatiashvili, Ana Westenberger, Anastasia Illarionova, Brit Mollenhauer, Christine Klein, Eva-Juliane Vollstedt, Franziska Hopfner, Günter Höglinger, Harutyun Madoev, Joanne Trinh, Katja Lohmann, Lara M. Lange, Manu Sharma, Sergiu Groppa, Thomas Gasser, Zih-Hua Fang, Karl Heilbron, Theresa Luth, Wenhua Sun, Inke König, Daniela Berg, Bernhard Haslinger, Teresa Kleinz, Norbert Brüggemann, Albert Akpalu, Georgia Xiromerisiou, Georgios Hadjigorgiou, Ioannis Dagklis, Ioannis Tarnanas, Leonidas Stefanis, Maria Stamelou, Efthymios Dadiotis, Alex Medina, Germaine Hiu-Fai Chan, Nancy Ip, Nelson Yuk-Fai Cheung, Phillip Chan, Xiaopu Zhou, Asha Kishore, Divya KP, Pramod Pal, Prashanth Lingappa Kukkle, Roopa Rajan, Rupam Borgohain, Mehri Salari, Tamara Shiner, Avner Thaler, Andrea Quattrone, Enza Maria Valente, Grazia Annesi, Lucilla Parnetti, Micol Avenali, Monica Gagliardi, Tommaso Schirinzi, Caterina Galandra, Anna De Rosa, Manabu Funayama, Nobutaka Hattori, Tomotaka Shiraishi, Altynay Karimova, Gulnaz Kaishibayeva, Aigerim Utegenova, Aigul Yemagambetova, Vadim Akhmetzhanov, Seitzhan Aidarov, Tautanova Raushan, Bagyzhan Syzdykova Syzdykova, Dinara Alzhanova, Zhanybek Myrzayev, Saltanat Abdraimova, Cholpon Shambetova, Rejko Krüger, Patrick May, Ai Huey Tan, Azlina Ahmad-Annuar, Mohamed Ibrahim Norlinah, Nor Azian Abdul Murad, Shahrul Azmin, Shen-Yang Lim, Wael Mohamed, Yi Wen Tay, Azalea Tenerife Pajo, Daniel Martinez-Ramirez, Mayela Rodriguez-Violante, Paula Reyes-Pérez, Alejandra Medina Rivera, Nancy Monroy Jaramillo, Bayasgalan Tserensodnom, Rajeev Ojha, Wilma Van De Berg, Bas Bleom, Bart Van de Warrenburg, Lisette Charbonnier, Tim J. Anderson, Toni L. Pitcher, Arinola Sanyaolu, Njideka Okubadejo, Oluwadamilola Ojo, Simon Izuchukwu Ozomma, Kolawole Wahab, Lasse Pihlstrøm, Manuela Tan, Ingeborg Haugesag Lie, Jodi Maple-Grødem, Shoaib Ur-Rehman, Mario Cornejo-Olivas, Maria Leila Doquenia, Raymond Rosales, Angel Vinuela, Elena Iakovenko, Anna Gareeva, Gulnara Akhmadeeva, Irina Gilyazova, Bashayer Al Mubarak, Muhammad Umair, Eng-King Tan, Jia Nee Foo, Elaine Chew, Vesna van Midden, Ferzana Amod, Jonathan Carr, Soraya Bardien, Nikita Pillay, Kathryn Step, Beomseok Jeon, Yun Joong Kim, Jung Hwan Shin, Joowon Jang, Esther Cubo, Ignacio Alvarez, Janet Hoenicka, Katrin Beyer, Maria Teresa Periñan, Pau Pastor, Ruben Fernandez-Santiago, Pilar Gómez Garre, Pablo Mir, Sarah El-Sadig, Kajsa Brolin, Per Svenningsson, Maria Swanberg, Christiane Zweier, Gerd Tinkhauser, Paul Krack, Chin-Hsien Lin, Hsiu-Chuan Wu, Pin-Jui Kung, Ruey-Meei Wu, Yihru Wu, Ganieva Manizha, Rim Amouri, Samia Ben Sassi, A. Nazlı Başak, Gencer Genc, Özgür Öztop Çakmak, Sibel Ertan, Alastair Noyce, Alejandro Martínez-Carrasco, Anette Schrag, Anthony Schapira, Camille Carroll, Donald Grosset, Eleanor J. Stafford, Henry Houlden, Huw R. Morris, John Hardy, Kin Ying Mok, Mie Rizig, Nicholas Wood, Nigel Williams, Olaitan Okunoye, Rauan Kaiyrzhanov, Rimona Weil, Seth Love, Simona Jasaityte, Sumit Dey, Vida Obese, Spencer Finch, Valentina Escott-Price, Hamin Lee, Roger Barker, Mina Ryten, Michele Hu, Laura Parkkinen, Kailash Bhatia, Richard Walker, Steve Gentleman, Thomas Warner, David Burn, Christian Lambert, Caroline Williams-Gray, Chris Morris, Deborah Attuah, Raquel Real, Yen Tai, Alberto Espay, Alyssa O’Grady, Andrew B. Singleton, Andrew K. Sobering, Bernadette Siddiqi, Bradford Casey, Brian Fiske, Cabell Jonas, Carlos Cruchaga, Caroline B. Pantazis, Charisse Comart, Claire Wegel, Cornelis Blauwendraat, Dan Vitale, Deborah Hall, Dena Hernandez, Ejaz Shiamim, Ekemini Riley, Faraz Faghri, Geidy E. Serrano, Hampton Leonard, Hirotaka Iwaki, Honglei Chen, Ignacio F. Mata, Ignacio Juan Keller Sarmiento, Jared Williamson, Jonggeol Jeff Kim, Joseph Jankovic, Joshua Shulman, Justin C. Solle, Kaileigh Murphy, Kamalini Ghosh Galvelis, Karl Kieburtz, Kate Andersh, Katerina Markopoulou, Kenneth Marek, Kristin S. Levine, Lana M. Chahine, Laura Ibanez, Laurel Screven, Lauren Ruffrage, Lisa Shulman, Luca Marsili, Maggie Kuhl, Marissa Dean, Mary B. Makarious, Matthew Farrer, Mathew Koretsky, Megan J. Puckelwartz, Miguel Inca-Martinez, Mike A. Nalls, Naomi Louie, Niccolò Emanuele Mencacci, Roger Albin, Roy Alcalay, Ruth Walker, Sara Bandres-Ciga, Sohini Chowdhury, Sonya Dumanis, Steven Lubbe, Tao Xie, Tatiana Foroud, Thomas Beach, Todd Sherer, Yeajin Song, Dana Lewis, Shreya Menon, Melissa Nirenberg, Spencer Grant, Shannon Ballard, Chad Shaw, Sidra Aslam, Geidy Serrano, Devin Sharp, Kensuke Daida, Rachel Saunders-Pullman, Michiko Kimura Bruno, Matt Farrer, Haydeh Payami, Ryan Pfingst, James B. Leverenz, Elizabeth Disbrow, Debi Brooks, Randy Schekman, Un Kang, Zbigniew K. Wszolek, Cyrus Zabetian, Zach Chaney, Christine Swanson-Fischer, Conor Hennessey, Cassandra Barrett, Beate Ritz, Bradley Boeve, Ashley Rawls, Duan Nguyen, Toan Nguyen, Masharip Atadzhanov, Hampton Leonard, Kajsa Atterling Brolin

**Affiliations:** 1 Laboratorio Internacional de Investigación Sobre el Genoma Humano, Universidad Nacional Autónoma de México, Juriquilla, Mexico; 2 Division of Neurology, Department of Medicine, Faculty of Medicine, University of Malaya, Kuala Lumpur, Malaysia; 3 Mah Pooi Soo & Tan Chin Nam Centre for Parkinson’s & Related Disorders, Faculty of Medicine, University of Malaya, Kuala lumpur, Malaysia; 4 Department of Anatomy, College of Medicine, University of Lagos, Lagos, Nigeria; 5 Coalition for Aligning Science, Washington, WA USA; 6 Genomic Medicine, Lerner Research Institute, Cleveland Clinic, Cleveland, OH USA; 7 Center for Alzheimer’s and Related Dementias (CARD), National Institute on Aging and National Institute of Neurological Disorders and Stroke, National Institutes of Health, Bethesda, MD USA; 8 Department of Clinical and Movement Neurosciences and Movement Disorders Centre, UCL Queen Square Institute of Neurology, University College London, London, UK; 9 DataTecnica, Washington, DC USA; 10 The Neuro (Montreal Neurological Institute-Hospital), McGill University, Montreal, QC Canada; 11 Department of Neurology and neurosurgery, McGill University, Montréal, QC Canada; 12 Department of Specialized Medicine, Division of Medical Genetics, McGill University Health Centre, Montreal, QC Canada; 13 Translational Neurogenetics Unit, Department of Experimental Medical Science, Lund University, Lund, Sweden; 14 Preventive Neurology Unit, Centre for Prevention, Detection and Diagnosis, Wolfson Institute of Population Health, Queen Mary University of London, EC1M 6BQ, London, UK; 15 Centro de Trastornos del Movimiento (CETRAM), Facultad de Ciencias Médicas, Universidad de Santiago de Chile, Santiago, Chile; 16 Fundación Parkinson Puerto Rico, Puerto Rico, USA; 17 Fundación Valle del Lili, Cali, Colombia; 18 Hospital Ángel C, Padilla, Argentina; 19 Facultad de Medicina, Universidad Nacional de Tucumán, Tucumán, Argentina; 20 Hospital das Clínicas, Faculdade de Medicina, Universidade de São Paulo, São Paulo, Brazil; 21 Departamento de Genética, Universidade Federal do Rio Grande do Sul, Porto Alegre, Brazil; 22 Universidade Federal de Ciências da Saúde de Porto Alegre, Porto Alegre, Brazil; 23 Hospital de Clínicas de Porto Alegre, Porto Alegre, Brazil; 24 Clínica Santa María, Santiago, Chile; 25 Faculdade de Medicina de Ribeirão Preto, Universidade de São Paulo, Ribeirão Preto, Brazil; 26 Hospital das Clínicas de Ribeirão Preto, Faculdade de Medicina de Ribeirão Preto, Universidade de São Paulo, Ribeirão Preto, Brazil; 27 Hospital das Clínicas de Ribeirão Preto, Universidade de São Paulo, Ribeirão Preto, Brazil; 28 Universidad Tecnológica Centroamericana (UNITEC), Tegucigalpa, Honduras; 29 Hospital de Especialidades San Felipe, Tegucigalpa, Honduras; 30 Hospital Interzonal General de Agudos “Gral. San Martín”, La Plata, Argentina; 31 Universidade Federal do Pará, Belém, Brazil; 32 Hospital Ophir Loyola, Belém, Brazil; 33 Hospital PUC-Campinas, Campinas, Brazil; 34 Hospital JM Ramos Mejía, Buenos Aires, Argentina; 35 Sanatorio de la Trinidad Mitre, Buenos Aires, Argentina; 36 Universidade Federal do Ceará, Fortaleza, Brazil; 37 Hospital Universitário Walter Cantídio, Universidade Federal do Ceará, Fortaleza, Brazil; 38 Universidad Peruana Los Andes, Huancayo, Peru; 39 Universidad Nacional del Altiplano, Puno, Peru; 40 Hospital Hipólito Unanue de Tacna, Tacna, Peru; 41 EDMECON Continuing Medical Education, Lima, Peru; 42 Universidad Científica del Sur, Lima, Peru; 43 Instituto Nacional de Neurología y Neurocirugía Manuel Velasco Suárez, Mexico City, Mexico; 44 Tecnológico de Monterrey, Escuela de Medicina y Ciencias de la Salud, Monterrey, Mexico; 45 Universidad de Antioquia, Medellín, Colombia; 46 Universidad de Chile, Santiago, Chile; 47 Hospital Clínico de la Universidad de Chile, Santiago, Chile; 48 Duke University School of Medicine, Durham, NC USA; 49 Hospital San Juan de Dios, San José, Costa Rica; 50 Universidad de Costa Rica, San José, Costa Rica; 51 Universidad Dr. Andrés Bello, San Salvador, El Salvador; 52 Instituto de Neurobiología, Universidad Nacional Autónoma de México, Querétaro, Mexico; 53 Universidad Nacional Autónoma de México, Mexico City, Mexico; 54 Facultad de Psicología, Universidad Nacional Autónoma de México, Mexico City, Mexico; 55 Centro de Ciencias Genómicas, Universidad Nacional Autónoma de México, Cuernavaca, Mexico; 56 Universidad Autónoma de Baja California, Tijuana, Mexico; 57 Universidad Autónoma de Querétaro, Querétaro, Mexico; 58 Neurociencias PRISMA AC, San Luis Potosí, Mexico; 59 Facultad de Medicina, Universidad Autónoma de San Luis Potosí, San Luis Potosí, Mexico; 60 Hospital Ángeles Clínica Londres, Mexico City, Mexico; 61 ISSSTE Morelia, Morelia, Michoacan, Mexico; 62 Hospital Civil de Guadalajara Fray Antonio Alcalde, Guadalajara, Mexico; 63 Centro Médico ABC, Mexico City, Mexico; 64 Hospital Ángeles Universidad, Mexico City, Mexico; 65 Private practice, Mexico City, Mexico; 66 University Hospital “Dr. José E. González”, Universidad Autónoma de Nuevo León, Monterrey, Mexico; 67 Hospital Ángeles Puebla, Puebla, Mexico; 68 Universidad Militar Nueva Granada, Bogotá, Colombia; 69 Universidad Nacional de Colombia, Bogotá, Colombia; 70 Universidad de la República, Montevideo, Uruguay; 71 Universidade Federal de São Paulo, São Paulo, Brazil; 72 University of Miami Miller School of Medicine, Miami, FL USA; 73 Xiangya Hospital, Changsha, China; 74 Capital Medical University, Beijing, China; 75 Zhejiang University, Hangzhou, China; 76 Xiangya Hospital, Central South University, Changsha, China; 77 University of Antioquia, Medellin, Colombia; 78 University of Costa Rica, San Jose, Costa Rica; 79 Aarhus University, Aarhus, Denmark; 80 The American University in Cairo, Cairo, Egypt; 81 Beni-Suef University, Beni Suef, Egypt; 82 Addis Ababa University, Addis Ababa, Ethiopia; 83 Paris Brain Institute, Paris, France; 84 Sorbonne Université, Paris, France; 85 Salpêtriere Hospital (AP-HP), Sorbonne Université, Paris, France; 86 Tbilisi State Medical University, Tbilisi, Georgia; 87 S. Khechinashvili University Hospital, Tbilisi, Georgia; 88 University of Lübeck, Lübeck, Germany; 89 Deutsches Zentrum für Neurodegenerative, Göttingen, Germany; 90 University Medical Center Göttingen, Göttingen, Germany; 91 University of Lübeck and University Medical Center, Lübeck, Germany; 92 Department of Neurology, University Hospital, LMU, Munich, Germany; 93 University of Tubingen, Tübingen, Germany; 94 University of Mainz, Mainz, Germany; 95 University of Tübingen, Tübingen, Germany; 96 The German Center for Neurodegenerative, Göttingen, Germany; 97 Charité - Universitätsmedizin Berlin, Berlin, Germany; 98 University of Luebeck, Lübeck, Germany; 99 University Medical Center Schleswig-Holstein, Lübeck, Germany; 100 Technical University of Munich, Munich, Germany; 101 University of Ghana Medical School, Accra, Ghana; 102 University of Thessaly, Volos, Greece; 103 Aristotle University of Thessaloniki, Thessaloniki, Greece; 104 Ionian University, Corfu, Greece; 105 Biomedical research Foundation of the Academy of, Athens, Greece; 106 Diagnostic and Therapeutic Centre HYGEIA, Marousi, Greece; 107 Hospital San Felipe, Tegucigalpa, Honduras; 108 Queen Elizabeth Hospital, Kowloon, Hong Kong; 109 The Hong Kong University of Science and, Kowloon, Hong Kong; 110 Aster Medcity, Kochi, India; 111 Sree Chitra Tirunal Institute for Medical Sciences, Thiruvananthapuram, India; 112 National Institute of Mental Health & Neurosciences, Bengaluru, India; 113 Manipal Hospital, Delhi, India; 114 All India Institute of Medical Sciences, Delhi, India; 115 Nizam’s Institute Of Medical Sciences, Hyderabad, India; 116 Shahid Beheshti University of Medical Science, Tehran, Iran; 117 Tel Aviv Sourasky Medical Center, Tel Aviv-Yafo, Israel; 118 Magna Græcia University of Catanzaro, Catanzaro, Italy; 119 University of Pavia, Pavia, Italy; 120 National Research Council, Cosenza, Italy; 121 University of Perugia, Perugia, Italy; 122 Magna Graecia University, Catanzaro, Italy; 123 University of Rome Tor Vergata, Rome, Italy; 124 IRCCS Mondino Foundation, Pavia, Italy; 125 University of Naples Federico II, Naples, Italy; 126 Juntendo University, Tokyo, Japan; 127 Juntendo University faculty of medicine, Tokyo, Japan; 128 Jikei University School of Medicine, Tokyo, Japan; 129 Institute of Neurology and Neurorehabilitation, Almaty, Kazakhstan; 130 West Kazakhstan Marat Ospanov State Medical Univ, Aktobe, Kazakhstan; 131 Medline medical center, Astana, Kazakhstan; 132 National Center for Neurosurgery, Astana, Kazakhstan; 133 Astana Medical University, Astana, Kazakhstan; 134 City multi-field hospital No. 1, Astana, Kazakhstan; 135 International University of Postgraduate Education, Almaty, Kazakhstan; 136 South Kazakhstan Medical Academy, Shymkent, Kazakhstan; 137 Kyrgyz State Medical Academy, Bishkek, Kyrgyzstan; 138 University of Luxembourg, Esch-sur-Alzette, Luxembourg; 139 University of Malaya, Kuala Lumpur, Malaysia; 140 Universiti Kebangsaan Malaysia, Selangor, Malaysia; 141 UKM Medical Molecular Biology Institute, Kuala Lumpur, Malaysia; 142 Universiti Kebangsaan Malaysia Medical Centre, Kuala Lumpur, Malaysia; 143 International Islamic University, Kuala Lumpur, Malaysia; 144 Tecnologico de Monterrey, Monterrey, Mexico; 145 Instituto Nacional de Neurologia y Neurocirugia, Mexico City, Mexico; 146 Universidad Nacional Autónoma de México, Santiago de, Mexico; 147 Mongolian National University of Medical Sciences, Ulaanbaatar, Mongolia; 148 Tribhuvan University, Kirtipur, Nepal; 149 Vanderbilt University Medical Center, Amsterdam, Netherlands; 150 Radboud University, Nijmegen, Netherlands; 151 Radboud University Medical Center, Nijmegen, Netherlands; 152 Brain Research and Innovation Center, Amsterdam, Netherlands; 153 University of Otago, Dunedin, New Zealand; 154 University of Lagos, Lagos, Nigeria; 155 College of Medicine of the University of Lagos, Lagos, Nigeria; 156 University of Calabar Teaching Hospital, Calabar, Nigeria; 157 University of Ilorin, Ilorin, Nigeria; 158 Oslo University Hospital, Oslo, Norway; 159 Stavanger University Hospital, Stavanger, Norway; 160 University of Science and Technology Bannu, Bannu, Pakistan; 161 Universidad Cientifica del Sur, Lima, Peru; 162 Metropolitan Medical Center, Manila, Philippines; 163 University of Puerto Rico, San Juan, Puerto Rico; 164 Research Center of Neurology, Moscow, Russia; 165 Ufa Federal Research Center, Ufa, Russia; 166 Ufa Scientific Center, Ufa, Russia; 167 Russian Academy of Sciences / Bashkir State Medica, Ufa, Russia; 168 King Faisal Specialist Hospital and Research Center, Riyadh, Saudi Arabia; 169 King Abdullah International Medical Research, Jeddah, Saudi Arabia; 170 National Neuroscience Institute, Singapore, Singapore; 171 Nanyang Technological University, Singapore, Singapore; 172 Ljubljana University Medical Centre, Ljubljana, Slovenia; 173 University of KwaZulu-Natal, Durban, South Africa; 174 University of Stellenbosch, Stellenbosch, South Africa; 175 Stellenbosch University, Stellenbosch, South Africa; 176 University of the Western Cape, Bellville, South Africa; 177 Stellenbosch University, Cape Town, South Africa; 178 Seoul National University Hospital, Seoul, South Korea; 179 Yongin Severance Hospital, Seoul, South Korea; 180 Hospital Universitario Burgos, Burgos, Spain; 181 University Hospital Mutua Terrassa, Barcelona, Spain; 182 Institut de Recerca Sant Joan de Deu, Barcelona, Spain; 183 Research Institute Germans Trias i Pujol, Barcelona, Spain; 184 Instituto de Biomedicina de Sevilla, Seville, Spain; 185 University Hospital Germans Trias i Pujol, Barcelona, Spain; 186 Hospital Clínic de Barcelona, Barcelona, Spain; 187 Faculty of medicine university of Khartoum, Khartoum, Sudan; 188 Lund University, Lund, Sweden; 189 Karolinska Institute, Stockholm, Sweden; 190 Inselspital Bern, University of Bern, Bern, Switzerland; 191 University Hospital Bern, Bern, Switzerland; 192 National Taiwan University Hospital, Taipei City, Taiwan; 193 Chang Gung Memorial Hospital, Taoyuan City, Taiwan; 194 Avicenna Tajik State Medical University, Dushanbe, Tajikistan; 195 National Institute Mongi Ben Hamida of Neurology, Tunis, Tunisia; 196 Mongi Ben Hmida National Institute of Neurology, Tunis, Tunisia; 197 Koç University, Istanbul, Turkey; 198 Şişli Etfal Training and Research Hospital, Istanbul, Turkey; 199 Queen Mary University of London, London, UK; 200 University of Plymouth, Plymouth, UK; 201 University of Glasgow, Glasgow, UK; 202 University College London, London, UK; 203 University of Bristol, Bristol, UK; 204 Cardiff University, Cardiff, UK; 205 St George’s, University of London, London, UK; 206 University of Oxford, Oxford, UK; 207 Northumbria Healthcare at NHS Foundation Trust, Newcastle upon Tyne, UK; 208 Imperial College London, London, UK; 209 Newcastle University, Newcastle upon Tyne, UK; 210 University of Cambridge, Cambridge, UK; 211 YLD, London, UK; 212 University of Cincinnati, Cincinnati, OH USA; 213 The Michael J. Fox Foundation for Parkinson’s, New York, NY USA; 214 National Institute on Aging, Bethesda, MD USA; 215 Augusta University / University of Georgia Medical, Augusta, GA USA; 216 Mid-Atlantic Permanente Medical Group, Bethesda, MD USA; 217 Washington University, St. Louis, MO USA; 218 National Institutes of Health, Bethesda, MD USA; 219 Indiana University, Bloomington, IN USA; 220 Rush University, Chicago, IL USA; 221 Kaiser Permanente, Oakland, CA USA; 222 Banner Sun Health Research Institute, Sun City, AZ USA; 223 Data Tecnica International, Washington, WA USA; 224 Michigan State University, East Lansing, MI USA; 225 Northwestern University, Evanston, IL USA; 226 Baylor College of Medicine, Houston, TX USA; 227 Parkinson’s Foundation, Princeton, NJ USA; 228 Beth Israel Deaconess Medical Center, Boston, MA USA; 229 North Shore University Health System, Chicago, IL USA; 230 Institute for Neurodegenerative Disorders, New Haven, CT USA; 231 University of Pittsburgh, Pittsburgh, PA USA; 232 University of Alabama at Birmingham, Birmingham, AL USA; 233 University of Maryland, Baltimore, MD USA; 234 University of Florida - Neurology, Gainesville, FL USA; 235 Cleveland Clinic, Cleveland, OH USA; 236 Universit of Michigan, Ann Arbor, MI USA; 237 Columbia University, New York, NY USA; 238 James J. Peters Veterans Affairs Medical Center, New York, NY USA; 239 Aligning Science Across Parkinson’s, Washington, WA USA; 240 Northwestern University, Chicago, IL USA; 241 University of Chicago, Chicago, IL USA; 242 Indiana University School of Medicine, Indianapolis, IN USA; 243 Sun Health Research Institution, Sun City, AZ USA; 244 Aligning Science Across Parkinson’s, Baltimore, MD USA; 245 Gladstone Institutes, San Francisco, CA USA; 246 Icahn School of Medicine at Mount Sinai, New York, NY USA; 247 Baylor College of Medicine / Texas Children’s, Houston, TX USA; 248 Banner Health, Phoenix, AZ USA; 249 Aligning Science Across Parkinson’s, Vancouver, BC USA; 250 The Queen’s Medical Center, Honolulu, HI USA; 251 University of Florida College of Medicine, Gainesville, FL USA; 252 The University of Alabama at Birmingham Heersink, School Birminghamof Medicine, Birmingham, AL USA; 253 LSU Health Shreveport, Shreveport, LA USA; 254 University of California, Berkeley, Berkeley, CA USA; 255 NYU Grossman School of Medicine, New York, NY USA; 256 Mayo Clinic College of Medicine, Rochester, MN USA; 257 VA Puget Sound Health Care System, Seattle, WA USA; 258 University of California, Los Angeles, Los Angeles, CA USA; 259 Mayo Clinic, Rochester, MN USA; 260 College of Medicine, University of Florida, Gainesville, USA; 261 Hue University, Hue, Vietnam; 262 University of Zambia, Lusaka, Zambia

**Keywords:** Diseases, Genetics, Neurology, Neuroscience

## Abstract

Variation at the 17q21.31 locus, which contains the gene encoding microtubule-associated protein tau (*MAPT*), has been associated with neurodegenerative disorders, including Parkinson’s disease (PD). This highly complex locus is characterized by two broadly defined haplotypes: H1 and the inverted H2 haplotype. While H1 has been associated with an increased PD risk and is present in all ancestry populations, H2 is enriched in individuals of European ancestry. So far, few studies have explored the H1 association with PD in non-European ancestries. Here, we investigated the haplotype and subhaplotype frequencies of H1 and H2 in 20,507 PD patients and 11,841 controls across eleven different ancestry groups from the Global Parkinson’s Genetics Program (GP2) and the Latin American Research consortium on the GEnetics of Parkinson’s Disease (LARGE-PD). Our results strongly support the involvement of the H1 haplotype in PD risk in individuals of European ancestry, with additional evidence suggesting an association across diverse ancestry groups. Additionally, we observed significant variation in the H1 subhaplotype frequencies within populations, highlighting the complexity of this genomic region and the relevance of its study in diverse ancestries to gain a more comprehensive understanding of the role this locus plays in neurodegenerative disease risk.

## Introduction

Parkinson’s disease (PD) is an age-related progressive neurodegenerative disorder marked by clinical heterogeneity and driven by a complex interplay of genetic and environmental factors that vary across regions and contexts, influencing both its development and progression^1,2^. Advances in the genetics of PD, particularly through large genome-wide association studies (GWAS) and meta-analyses, have identified numerous risk loci and candidate genes^3–5^. Although many of these studies have predominantly focused on populations of European ancestry, efforts are increasing to include underrepresented non-European populations^6–9^. Among the loci identified, the 17q21.31 region has been of interest due to its consistent association in European cohorts and its complex genomic architecture.

In 2009, Simón-Sánchez and colleagues conducted a European GWAS in PD, including 5074 patients and 8551 controls, and identified two association signals linked to PD risk: one in *SNCA* and the other at the 17q21.31 locus^10^ and 17q21.31 locus has been consistently replicated in subsequent GWAS performed in European ancestry participants^5^. However, also in 2009, Satake and colleagues conducted a PD GWAS in individuals of Asian ancestry, including 2011 patients and 18,381 controls, and did not find the association with variability at the 17q21.31 locus^11^. This association also failed to be detected in the more recent and larger GWAS of South East Asian ancestry participants by Foo et al. ^12^.

*MAPT*, located on chromosome 17q21.31, encodes the microtubule-associated protein tau, and consists of 15 exons, and is primarily expressed in neurons, where it regulates axonal transport^13–15^. The genomic architecture of the 17q21.31 region is highly complex, featuring a ~1.8 Mb block of linkage disequilibrium (LD) defined by a 900 kb inversion and characterized by two main haplotypes, H1 and H2^13,14,16^. This locus represents the largest area of LD known in the human genome, and the inversion spans 15 genes as well as several non-coding RNAs and pseudogenes^17,18^. The H1 haplotype exhibits normal genetic variation patterns, leading to multiple subhaplotypes^14^. Single nucleotide variants (SNVs) can distinguish H1 from H2, with H1-specific SNVs generating several common H1 subhaplotypes^16^. Numerous SNVs can be used, but the most commonly used for haplotype definition are rs8070723 and rs1052553, while sub-haplotype definitions are often based on rs1467967, rs242557, rs3785883, rs2471738, and rs7521, all located in *MAPT*^18^.

The H1 haplotype is observed in all populations, whereas the H2 haplotype (inverse orientation) is enriched in individuals of European ancestry, with a prevalence of about 20%. It is less common in African ancestry populations and nearly absent in East Asian ancestry populations, and shows very limited genetic variability^14,17–19^. The H1 haplotypes have been associated with an increased risk for several synucleinopathies, including PD, dementia with Lewy bodies, and multiple system atrophy^14,16,20–22^. In contrast, the H2 haplotype is thought to reduce the risk for these neurodegenerative diseases^4,14,16,23^. The H1 haplotype and H1c sub-haplotype are also strongly associated with Progressive Supranuclear Palsy (PSP), a primary tauopathy which involves the deposition of four-repeat predominant tau protein^24^. There is some evidence that H1 and H1c increase the expression of *MAPT* and the inclusion of exon 10, providing a mechanistic link between the genetic risk and PSP neuropathology^25,26^. However, many PD patients do not have tau pathology at post-mortem, and it is uncertain whether *MAPT* itself explains the association between PD and the H1 locus. In fact, the haplotypes and variants at the locus have been shown to regulate gene expression of other genes at the locus, i.e., *KANSL1*, *CRHR1*, and *LRRC37A/4*, in addition to *MAPT*^27,28^. Although the 17q21.3 locus risk association is a prominent finding in PD genetics, the H1/H2 association has been largely reported in European populations, likely related to the frequency and sample size of the non-European studies and the low H2 background allele frequency in Africa and Asia^17,29^. Given the limited research on 17q21.3 haplotypes in PD among non-European cohorts, this study aims to investigate the frequency and associations of the H1 and H2 haplotypes with PD across diverse ancestries using data from the Global Parkinson’s Genetic Program (GP2, https://gp2.org/)^30^ in 20,507 PD patients and 11,841 controls and the Latin American Research consortium on the Genetics of Parkinson’s Disease (LARGE-PD)^8^, consisting of 775 PD patients and 673 controls. Association analysis in non-European populations may help define the causal alleles, and by examining these haplotypes across a broad range of ancestry groups, we seek to enhance our understanding of their role in PD and provide new insights into the genetic underpinnings across diverse populations.

## Results

**Fig. 1:**
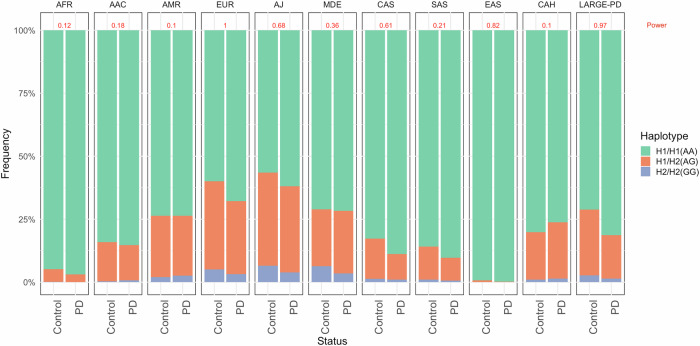
H1/H2 allele/haplotype frequency (rs1052553) in PD patients and controls in different ancestry groups. H1/H2 allele/haplotype frequency (rs1052553) in PD patients and controls in African (AFR), African American (AAC), Admixed American/Latin American (AMR), European (EUR), Ashkenazi Jews (AJ), Middle Eastern (MDE), Central Asian (CAS), South Asian (SAS), East Asian (EAS), Complex Admixture History (CAH) populations and the Latin American Research consortium on the GEnetics of Parkinson’s Disease (LARGE-PD). In red at the top of each plot, the power for each ancestry is shown.

The rs1052553-AA genotype, representing the H1/H1 haplotype, was the most frequent across all ancestry populations, followed by H1/H2 (AG) and H2/H2 (GG) (Fig. 1 and Supplementary Data 3). The H2 haplotype frequency varied across the ancestry groups, ranging from 0.1% in PD patients and 0.4% in controls in the EAS ancestry population to the highest frequencies in the AJ population, with 21.0% among PD patients and 25.0% in controls. In most groups, H2 was more common in controls than in PD patients, except in the AMR and CAH ancestry groups, where H2 was more common among PD patients, MAF_H2_ = 14.2% vs. 14.5% in AMR and MAF_H2_ = 10.5% vs. 12.6% in CAH. The rs1052553 variant was in HWE in the control group in all populations except for the MDE population (*p* = 0.004) (Supplementary Data 4).

A nominal statistically significant difference for the allele/haplotype frequency between PD patients and controls was observed in the five ancestry populations AFR (OR = 1.76, 95% confidence interval [CI] = 1.14–2.70, *p* = 0.0095), AJ (OR = 1.256, 95% CI = 1.04–1.52, *p* = 0.012), CAS (OR = 1.58, 95%CI = 1.07–2.30, *p* = 0.016), EAS (OR = 3.28, 95%CI = 1.30–8.27. *p* = 0.0077), and EUR (OR = 1.35, 95%CI = 1.28–1.43, *p* = 2.29E-26), as well as in the LARGE-PD cohort (OR = 1.67, 95%CI = 1.34–2.08, *p* = 4.6E-6) (Supplementary Data 5 and Supplementary Fig. 1).

**Fig. 2:**
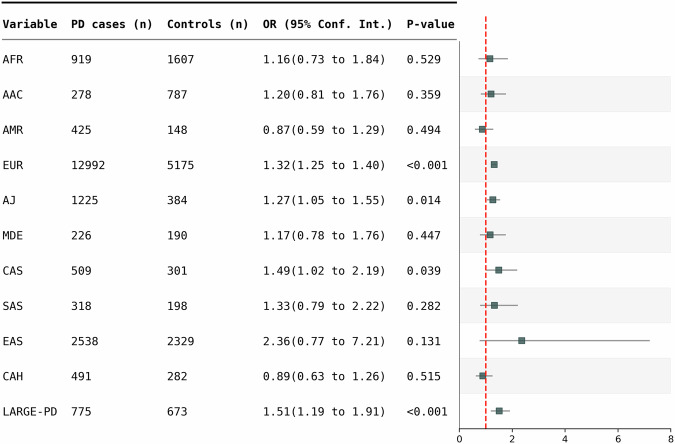
Forest plot showing odds ratio (OR) estimates with a 95% confidence interval for the *MAPT* containing H1 haplotype with Parkinson’s disease in the GP2 and LARGE-PD cohorts. *N* shows the sample size per population after adjusted regression (sex and PC1-5). A dashed red line indicates OR = 1. AFR African, AAC African American, AMR Admixed American/Latin American, EUR European, AJ Ashkenazi Jews, MDE Middle Eastern, CAS Central Asian, SAS South Asian, EAS East Asian, CAH Complex Admixture History. LARGE-PD Latin American Research consortium on the GEnetics of Parkinson's Disease.

Additionally, after adjusting the regression for age, sex, and PC1-5, a statistically significant association between the H1 haplotype and PD was only observed in the EUR population (OR = 1.33, 95%CI = 1.24–1.43, *p* = 2.4E-15) and the LARGE-PD cohort (OR = 1.51, 95%CI = 1.19–1.93, *p* = 0.000745) (Supplementary Data 6 and Supplementary Fig. 2). Complete-case analyses were used for the multivariate logistic regression, resulting in a substantial loss of participants in several ancestry groups due to missing age data. To increase the sample size and hence the statistical power, we additionally ran a multivariate logistic regression only including sex and PC1-5 as covariates which generated nominal significant associations in the AJ (OR = 1.27, 95%CI = 1.05–1.54, *p* = 0.0149) and CAS ancestries (OR = 1.57, 95%CI = 1.08–2.26, *p* = 0.0169), in addition to the significant associations in the EUR population (OR = 1.32, 95%CI = 1.25–1.30, *p* = 1.31E-22), and the LARGE-PD cohort (OR = 1.51, 95%CI = 1.19–1.91, *p* = 0.00067) (Supplementary Data 7 and Fig. 2).

No differences were observed for PD AAO between H1 and H2 haplotype carriers in any ancestry group (Supplementary Data 8). A nominal association (not passing Bonferroni significance) between the H1 haplotype and AAO was observed in a linear regression adjusted for sex and 5PCs in the AJ and LARGE-PD groups (Supplementary Data 9), with the H1 haplotype being associated with a ~ 1.3 and ~3.2 years earlier AAO compared to the H2 haplotype (AJ: beta = −1.368, SE = 0.657, *p* = 0.0374, LARGE-PD: beta = −3.175, SE = 1.253, *p* = 0.0115).

The subhaplotype analyses included six tagging variants, which capture the H2 haplotype along with the variation patterns of the H1 subhaplotypes. The variants were in HWE in all populations with a few exceptions (chr17:45908813:G:A in AFR, chr17:45942346:G:A in AJ, chr17:46003698:A:G in AAC and MDE, and chr17:46028029:A:G in LARGE-PD) (Supplementary Data 10). To investigate the accuracy of the genomic imputation for the data used in the subhaplotype analysis, we evaluated the subhaplotype pattern in a subset of individuals of the AFR and of the EUR ancestry group for which both WGS and imputed genotyping data were available. We observed a similar pattern for both methods, with minor allele frequency discrepancies (<0.4 percentage points difference in EUR and <2.1 percentage points in AFR [Supplementary Figs. 3 and 4]).

**Fig. 3:**
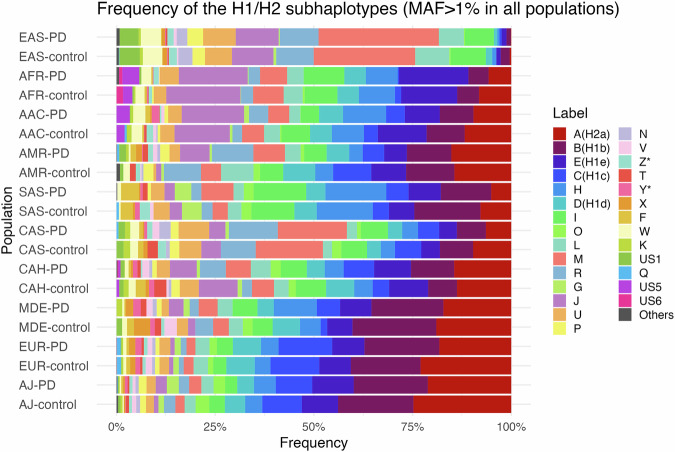
Frequency of H1 and H2 subhaplotypes in PD patients and controls. Showing the frequency (MAF > 1%) of the H1 and H2 subhaplotypes in different ancestry groups. Plotsots are ordered by the frequency of A(H2a) subhaplotype in controls. Subhaplotype names shown on the right panel correspond to Pittman SNV-based nomenclature. AFR African, AAC African American, AMR Admixed American/Latin American, EUR European, AJ Ashkenazi Jews, MDE Middle Eastern, CAS Central Asian, SAS South Asian, EAS East Asian, CAH Complex Admixture History, LARGE-PD Latin American Research consortium on the GEnetics of Parkinson's Disease. US Unspecified subhaplotype.

A large variation in the H1 subhaplotype frequencies was observed between several ancestry populations (Fig. 3 and Supplementary Data 11). EUR, AJ, and MDE populations showed a similar pattern, with H1b subhaplotype being the most frequent, followed by H1c. In contrast, these subhaplotypes were rare in the EAS population (1.6% and 0.4%, respectively), where H1 subhaplotype M was the most common, similar to the CAS population. The highest frequency of the H1J subhaplotype was seen in AFR (17.1%) and AAC populations (13.8%), followed by EAS (10.7%), with the lowest in the EUR population (1.1%). A statistically significant difference in the frequency between PD patients and controls was found only for H2a subhaplotype in EUR (*p* = 5.01E-24) and the four H1 subhaplotypes H1b (*p* = 0.0003), I (*p* = 0.0009), L (*p* = 1.6E-05), and M (*p* = 4.2E-07) in EAS after Bonferroni correction (*p* = 0.05/33 = 0.0015).

After ranking the subhaplotypes by Gini importance score, none of the subhaplotypes with the highest importance scores overlapped between the ancestries (Supplementary Data 12). In the EUR ancestry group, the H2A subhaplotype was ranked twice as important as the second and third most important subhaplotypes, the US6 and H1C subhaplotypes, further emphasizing that the H2a subhaplotype is most important in distinguishing between PD patients and controls in the EUR ancestry group. In the EAS ancestry group, the L subhaplotype ranked three times higher than the second-ranking haplotype (the Y* subhaplotype), indicating the importance of the L subhaplotype in this ancestry group.

For subhaplotypes in H2, the H2a (SNV-based nomenclature^31^) was seen to be the most common in all ancestry groups. The same SNV tagging the H2 (H2a) haplotypes in the analysis (rs8070723) has also been described to tag the H2D subhaplotype (CNV-based nomenclature^18^). Further analyses of the H2 haplotype showed that the H2D haplotype was most common in all ancestry groups with the exception of the AFR population, where the H2.1 subhaplotype was more frequent (Table 1).

**Table 1:** Frequency of the major H2 subhaplotypes in the GP2 ancestry populations and LARGE-PD

		AFR	AAC	AMR	EUR	AJ	MDE	CAS	SAS	EAS	CAH	LARGE-PD
H2’ (H2.1) frequency	All (%)	1.39	1.78	1.34	1.62	3.00	2.37	1.22	0.78	0.03	2.26	1.06
	Controls (%)	1.39	2.02	1.34	1.97	4.11	3.08	0.99	1.01	0.06	2.47	1.46
	PD patients (%)	1.40	1.08	1.41	1.48	2.65	1.76	1.35	0.63	NA	2.13	0.71
H2D frequency	All (%)	0.88	6.21	12.58	17.28	17.31	14.26	5.82	5.32	0.21	9.52	11.55
	Controls (%)	1.27	6.05	11.74	20.31	19.29	14.36	7.87	6.57	0.32	7.95	14.17
	PD patients (%)	0.22	6.65	12.90	16.07	16.69	14.18	4.62	4.54	0.12	10.42	9.22

*AFR* African, *AAC* African American, *AMR* Admixed American/Latin American, *EUR* European, *AJ* Ashkenazi Jews, *MDE* Middle Eastern, *CAS* Central Asian, *SAS* South Asian, *EAS* East Asian, *CAH* Complex Admixture History.

**Fig. 4:**
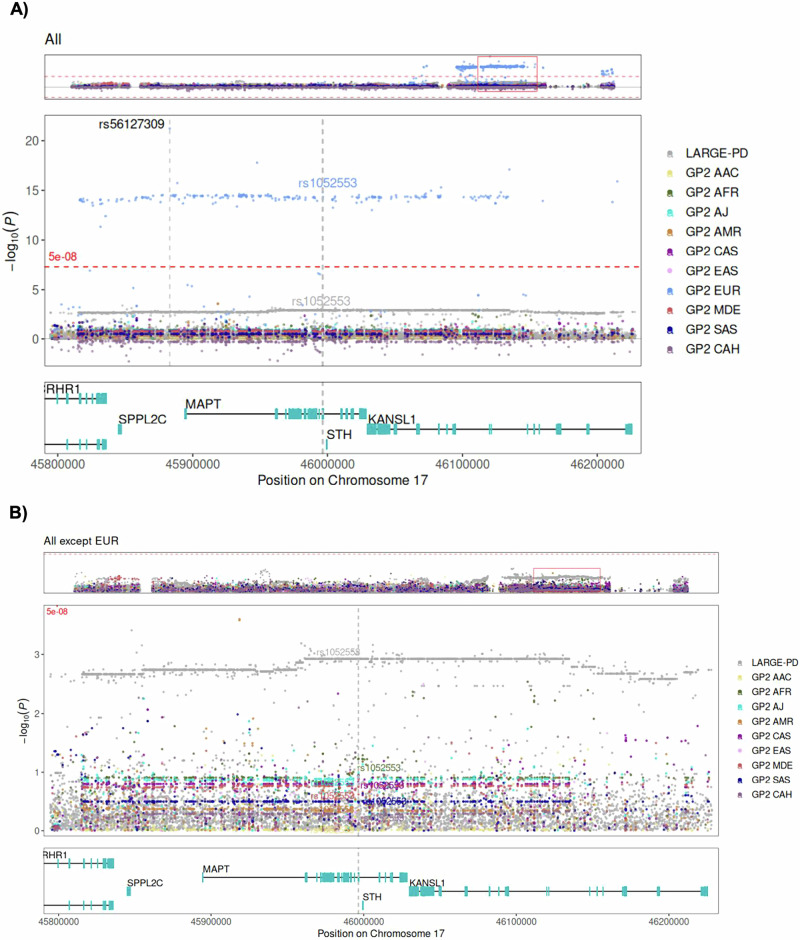
Regional plot of variant associations at the *MAPT* locus. **A** Variant association results across all ancestry groups from the GP2 and LARGE-PD cohorts. **B** The same analysis in which the EUR population was removed to improve visualization of associations in the remaining populations. Association results are derived from an adjusted regression model with MAF filter of >1%. The y-axis represents –log₁₀(p) values of variant associations, while the x-axis displays genomic positions. In each plot, the upper panel presents the full 17q21.31 genomic region (chr17:42,800,001–46,800,000, UCSC Genome Browser, GRCh38/hg38). The middle panel zooms in on the *MAPT* region (chr17:45,794,527–46,228,334, with significant variants annotated by their rs IDs. The H2-tagging SNV (rs1052553) is marked by a vertical gray dotted line. The lower panel displays protein-coding genes adjacent to *MAPT*. The horizontal red dashed line represents the genome-wide significance threshold. AFR African, AAC African American, AMR Admixed American/Latin American, EUR European, AJ Ashkenazi Jews, MDE Middle Eastern, CAS Central Asian, SAS South Asian, EAS East Asian, CAH Complex Admixture History. LARGE-PD Latin American Research consortium on the GEnetics of Parkinson's Disease.

We additionally generated regional plots to explore the association signals in the 17q21.31 region across the different ancestry groups. In the EUR population, which had a considerably larger sample size compared to other groups, we observed significant associations between multiple variants and PD throughout the region of *MAPT*. The pattern of significant variants is indicative of the strong LD in this region and a similar LD block structure was observable in the plots for the other ancestry populations; however, no significant association was observed between PD and any variant in the region at genome-wide significance level (Fig. 4 and Supplementary Fig. 4).

## Discussion

This study provides a comprehensive investigation of the *MAPT*-containing H1 and H2 haplotypes in PD across multiple ancestry groups. Our findings highlight the population-specific variability in haplotype frequencies and their association with PD risk, underscoring the importance of studying diverse cohorts in PD genetics. We observed that the H1 haplotype was present in all ancestry groups, while the H2 haplotype was significantly more frequent in EUR and AJ populations and nearly absent in the EAS ancestry population. This finding aligns with prior reports describing the H2 haplotype as a predominantly European marker with limited genetic variability^18,32,33^.

Earlier studies of the H1 haplotype and PD have yielded mixed results. Several European cohorts reported an association of the H1 haplotype with PD, including Greek (OR:1.6, 95% CI: 1.1–2.2;)^34^, Serbian (OR:2.0, 95% CI: 1.3–3.2)^35^, Norwegian (OR:1.9, 95% CI: 1.3–2.6)^36^ and U.S. samples of European descent (OR:1.5, 95%CI: 1.3–1.7)^5^, yet no signal was confirmed in a German cohort^35^. Additionally, GWAS in individuals of European^5,10^ and Latin American ancestries^8^ have identified variants in the 17q21.31 region as associated with PD. Also, a recent PD GWAS in the Indian population observed a borderline genome-wide significant signal at the *MAPT* locus with the H1/H2 haplotype tagging variant rs8070723 and identified a H2 haplotype frequency of 4% in the Indian population^37^. In contrast, no association was observed in a Mexican mestizo population^38^, in the Nigeria Parkinson’s Disease Research network cohort^29^ or in the Asian population GWAS^11,12^. Due to the complex LD structure, the *MAPT* locus was excluded in the multi-ancestry PD GWAS by Kim et al. ^9^ and the high LD makes it difficult to identify potential specific causal variants in the region^18^. Haplotype-based analyses are therefore essential to provide a more comprehensive understanding of the genetic variability in the region.

Our results suggest that the H1 haplotype is associated with PD in multiple ancestry groups, with an effect size ranging from OR = 1.27 to OR = 1.51, with the significance of the association varying in relation to the H2 allele frequency. We hypothesize that the previous report of a lack of association is due to the sample sizes and that, as the sample size from diverse ancestry groups grows in GP2, the H1 haplotype will show an association with PD in all populations. Supporting this is the observation of the H1 haplotype being more common among PD patients than controls in all ancestry groups with the exception of the GP2-AMR and the CAH ancestries (with a similar MAF for PD patients and controls). The conclusions that can be drawn from the CAH ancestry group are restricted by the limited sample size (491 PD patients and 282 controls), and the CAH ancestry group consists of highly admixed individuals from various backgrounds, which would require special consideration and analysis to disentangle the genetic architecture of this locus. Due to varying MAF in the different ancestry groups, sufficient power to detect a true association was only estimated for EUR, EAS, and LARGE-PD. To forecast the number of PD patients and controls needed to achieve 80% power in the different ancestry groups, we performed power calculations assuming a similar effect size as the EUR ancestry group (OR = 1.32). As an example, in the AFR ancestry group, where the frequency of the H2 haplotype is ~2.1%, we estimate that we would need ~4360 PD patients and the same number of controls to reach significance at *p* = 0.05.

We show that there is much greater haplotypic diversity across the 17q21.31 locus in non-European population groups, enabling fine mapping of functional alleles in different populations. While the H2 haplotype is most common in European populations, different H1 subhaplotypes, notably H1J and H1M, are more common in AFR and EAS, respectively. A potential association was only observed for four subhaplotypes in the EAS ancestry group, with the L subhaplotype ranking as the most important feature, whereas the H2a subhaplotype had the highest rank in the EUR ancestry group. This could give us an indication of the subhaplotypes of highest importance in PD, but further analyses are needed to fully elucidate this. We additionally show that the H2D haplotype was most common in all ancestry groups with the exception of the AFR population, where the H2’ (H2.1) subhaplotype was more frequent (although at low MAF), which is likely an ancestral H2 haplotype and has previously been reported to be enriched in African hunter-gatherer groups as compared to West Africans populations^39^.

The four major sub-haplotypes of H1 and H2 have been described to be H1’, H1D, H2’, and H2D based on the inversion and copy number status of *KANSL1*^39^. H1’ and H2’ carry no duplications (also defined as H2.1), whereas H1D spans a 205-kb duplication and H2D a shorter (155-kb) duplication. As CNVs were not investigated here and no common SNVs have been identified that could distinguish H1′ from H1D haplotypes^39^, we relied on the SNV-based nomenclature from Pittman et al. to distinguish H1 subhaplotypes^31^. However, for H2, this method only describes the H2a subhaplotype, and the same tag SNVs also tag the H2D subhaplotype^18^. In contrast to H1D, the H2D can be defined based on multiple SNVs in the region, while other SNVs located in *KANSL1* describe the H2’ subhaplotype. Pedicone and colleagues have suggested a hybrid nomenclature for subhaplotypes using Steinberg CNV-based nomenclature merged with Pittman SNV-based nomenclature, separated by underscore^18^. As the authors note, CNV- and SNV-based nomenclatures do not completely overlap and should be considered independently. Due to the absence of CNV data for H1, we relied solely on SNV-based methods.

Our study has several limitations. Firstly, although the study encompasses a substantial and diverse dataset, some demographic groups are still underrepresented, which may affect the applicability of the findings across all populations. Additionally, using other methods in the future, such as SHAPEIT5, would be of interest to estimate subhaplotypes in a more unbiased approach, with better estimates of rare variants^40^. We also observed missing data on key covariates, such as age, in a significant portion of individuals, which reduced the sample size and analytical robustness, resulting in the results being reported without adjusting for age. Moreover, our subhaplotype analysis is based on imputed genotyping data. While this method is generally reliable, it can introduce some uncertainty in haplotype determination, especially in populations with less comprehensive linkage disequilibrium reference panels. To mitigate this issue, we compared results with WGS data, which revealed only minimal discrepancies. In addition, occasionally, the investigated variants deviated from HWE. However, inspection of genotype quality metrics did not reveal evidence of poor variant quality or batch-specific effects, thus this deviation is believed to be a consequence of the structural complexity of this region^18,41^. Furthermore, analyses were conducted using datasets from GP2 and LARGE-PD cohorts, which employed different genotyping panels (NBA^42^ vs MEGA^8^), which may introduce inconsistencies in variant coverage and affect the comparability of results across cohorts. Finally, our study did not investigate CNVs, which are known to contribute to the genetic diversity of the 17q21.31 region and its association with PD^39^.

In conclusion, our study reinforces the significant role of the *MAPT*-containing H1 haplotype in PD across multiple populations. Our findings highlight the genetic diversity of 17q21.31 haplotypes across different populations. It further underscores the importance of including underrepresented populations in genetic analyses. Future research should focus on understanding the structural complexity of the 17q21.31 region, utilizing long-read sequencing and multi-omics approaches, and ensuring the inclusion of diverse populations to gain a more comprehensive understanding of *MAPT*-related neurodegenerative disease risk.

## Methods

### Samples

We investigated the frequency of the H1 and H2 haplotypes using genotyping data from the GP2 release 7 (https://zenodo.org/records/10962119)^43^, consisting of 20,507 PD patients and 11,841 controls from ten different ancestry groups: African American (AAC), African (AFR), Ashkenazi Jews (AJ), Admixed American/Latin American (AMR), Central Asian (CAS), Complex Admixture History (CAH), East Asian (EAS), European (EUR), Middle Eastern (MDE) and South Asian (SAS). Additionally, we investigated the haplotype frequencies in LARGE-PD^44^. The LARGE-PD cohort includes 775 Parkinson’s disease (PD) patients and 673 controls, recruited from nine clinical sites across South America, and represents a Latino or American admixed population comparable to the GP2-AMR cohort. Although the LARGE-PD cohort can be classified under the AMR population label, for clarity and ease of reading, we will refer to GP2-AMR as “AMR” and LARGE-PD as “LARGE-PD” throughout this work. Data was obtained in accordance with the ethical standards of the Helsinki Declaration. After filtering out related individuals and individuals with missing genotype data for the investigated H2-tagging SNV, a total of 19,921 PD patients and 11,401 controls remained for the analysis. The breakdown of the numbers of PD patients and controls is listed in Table 2, and a summary of demographics can be found in Supplementary Data 1.

**Table 2:** Number of PD patients and controls from each ancestry included in the study

Population	Sample size	
	PD patients	Controls
African American (AAC)	278	787
African (AFR)	919	1607
Ashkenazi Jews (AJ)	1225	384
Admixed American/Latin American (AMR)	425	148
Complex Admixture History (CAH)	491	282
Central Asian (CAS)	509	301
East Asian (EAS)	2538	2329
European (EUR)	12,992	5175
Middle Eastern (MDE)	226	190
South Asian (SAS)	318	198
The Latin American Research consortium on the GEnetics of Parkinson’s Disease (LARGE-PD)	775	673
**Total**	**19,921**	**11,401**

### Power calculation

Power calculations were done using the Genetic Association Study (GAS) Power Calculator (https://csg.sph.umich.edu/abecasis/cats/gas_power_calculator/) using an additive model, a significance level of *p* = 0.05, and a disease prevalence of 0.5% with the available sample sizes and the observed minor allele frequency (MAF) in each ancestry group, ranging from 0.26% in EAS to 22.99% in AJ. Additionally, the estimated number of samples needed to reach 80% power at the effect size (odds ratio [OR]) observed in the EUR population was calculated for the observed MAF in the different ancestry groups (Supplementary Data 2).

### Genetic data and analyses

Genotyping (Illumina NeuroBooster Array [NBA])^42^, sample and variant quality control (QC), imputation, ancestry prediction, and processing in GP2 were previously performed using GenoTools v1.0, and have been described elsewhere (https://github.com/dvitale199/GenoTools)^45^. All analyses using GP2 data were conducted on the cloud-native platform, Terra Community Workbench (https://app.terra.bio/), and were performed separately for all ancestry groups. The LARGE-PD data was genotyped using the Illumina Multi-Ethnic Genotyping Array [MEGA]^8^. Genotyping QC and relationship control were performed using the LARGE-PD QC pipeline (https://github.com/MataLabCCF/GWASQC). This pipeline was specifically developed for QC in admixed populations, minimizing sample loss. Imputation for both GP2 and LARGE-PD was done using the TOPMed reference panel as described^46^. While GP2 and LARGE-PD samples were genotyped using NBA and MEGA arrays, respectively, these platforms share the Infinium Global Diversity Array (GDA) backbone, and both datasets were imputed using the TOPMed imputation server and underwent similar quality control procedures. No evidence of batch effects attributable to the genotyping platform was observed based on principal components analysis.

17q21.3 H1/H2 haplotypes were investigated using the H2-tagging SNV rs1052553 (chr17:45996523:A:G) from the raw genotyping data according to the following allele combinations: AA representing the H1/H1 haplotype, AG the H1/H2 haplotype, and GG the H2/H2 haplotype. Allele and haplotype frequencies, Hardy Weinberg equilibrium (HWE), and ancestry-specific associations with PD risk and PD age at onset (AAO) were analyzed using PLINK v1.9 and v2.0 with and without adjusting for (i) sex and PC1-5 and (ii) sex, age, and PC1-5 (PD risk only). Multiple-testing correction for ancestry-specific H1/H2 analyses was performed using Bonferroni correction for the number of ancestry groups (*n* = 11), resulting in Bonferroni-corrected *p*-value of 0.05/11 = 0.0045. HWE was calculated in the control group for all ancestry populations, and standard genotype quality control metrics (call rate and imputation quality) were inspected for rs1052553. Plots for the frequencies across ancestries were done using *ggplot* in R version 4.1.2, while association results were visualized using the *forestplot* package in Python version 3.10.9. Potential differences for AAO between H1 and H2 carriers were evaluated using the Mann–Whitney U test, as normality was assessed by visual inspection of the data distributions, and AAO was not normally distributed. A linear regression adjusted for sex and PCs 1–5 was also performed.

Six tagging variants in the locus (chr17:45908813:G:A - rs1467967; chr17:45942346:G:A - rs242557; chr17:45977067:A:G - rs3785883; chr17:45998697:C:T - rs2471738; chr17:46003698:A:G - rs8070723; and chr17:46028029:A:G - rs7521) were used to construct the most common subhaplotypes as previously described using the Pittman SNV-based nomenclature^31,47^. While the Pittman subhaplotype nomenclature was originally derived using EM-based haplotype inference and phased trio data, in the present study, we did not aim to re-infer haplotype structure, but to assign individuals to these previously defined haplotype classes. Individual-level haplotypes were therefore not phased prior to analysis. These variants have been reported to capture 95% of the common 17q21.3 haplotype diversity in Europeans^31^, where five of these variants are H1-specific. To distinguish the H2 subhaplotypes (H2 and H2D), the SNVs rs1052553 (chr17:45996523:A:G - rs1052553, chr17:46724418:G:A - rs199451, and chr17:46751565:G:A - rs199533) were used^18,39^. We required that all three SNVs match the expected haplotype. Multiple testing for subhaplotypes was addressed using false discovery rate (FDR) correction according to the Benjamini–Hochberg procedure. FDR correction was applied per ancestry group, across all tested subhaplotypes within each analysis.

Since not all variants were captured during genotyping, we utilized multi-ancestry imputed genotyping data to investigate the H1 subhaplotypes. As genetic imputation uses known haplotypes in a reference population, we investigated the subhaplotypes in a subset of individuals where both whole genome sequencing (WGS) data (GP2, release 6^48^) and imputed genotype data were available in GP2 to evaluate the accuracy. This was evaluated in 150 individuals (99 PD patients and 51 controls) of the AFR ancestry group and 533 individuals (452 PD patients and 92 controls) of the EUR ancestry group.

To further characterize the relationship between 17q21.3 subhaplotypes and PD, we performed feature selection using the Munge module of GenoML (v1.5.4), an automated machine learning tool for genomics, in a per-ancestry analysis. This module ranks input features according to their potential contribution to prediction performance using scikit-learn’s Extra Trees Classifier. Subhaplotype importance is quantified using Gini importance, with higher values indicating a more informative feature for model predictions. To create the subhaplotype input, imputed pVCFs were first subset to the six aforementioned tagging SNVs and then recoded to create a PLINK.raw file where each row represents a single sample. Each SNV was coded as follows: 0, for being homozygous for the reference allele, 1 for heterozygous, and 2 for homozygous for the alternate allele. These values were then mapped to the corresponding SNVs’ alleles to form the subhaplotype labels. For example, a sample with all reference alleles (000000) would have the corresponding 17q21.3 subhaplotype: GGACAA, or A (H2a). A total of 26,312 samples out of the 31,322 included in the study were heterozygous for at least one tagging SNV. Due to the exploratory nature of our analysis, we did not investigate the effect of multiple subhaplotype copies, instead focusing on whether the presence of certain subhaplotypes was ranked as more important for predicting PD cases from controls in the Extra Trees Classifier. Heterozygosity at the tagging SNVs were coded as the alternate allele. After assigning a subhaplotype to each sample, the resulting data were converted into a binary representation using one-hot encoding for input into GenoML. Using the Munge module, the one-hot encoded subhaplotype file was set with the additional data parameter. Covariates (sex, PC1-5) and phenotypes were also added with the confounders and phenotype parameters, respectively. The code can be found on our GitHub (https://github.com/GP2code/MAPThaplotype/tree/main), and further use cases for GenoML at (https://github.com/GenoML/genoml2). The resulting output is a per-ancestry list of the input subhaplotypes, and their corresponding Gini importance score (Supplementary Table 12).

Additionally, to get a general view of the potential association between the 17q21.31 genomic region and PD, we extracted the region (chr17:42,800,001-46,800,000 [UCSC Genome Browser, GRCh38/hg38]) and conducted association and logistic regression analyses using PLINK v2.0, adjusting for sex, age, and the first 5 PCs, filtering by MAF > 1%. A regional plot spanning the 17q21.31 locus was visualized employing the *topr* tool version 1.1.8^49^ in R v4.4.0.

## Supplementary information

Supplementary Figures

Supplementary Data

## Data Availability

Data used in the preparation of this article were obtained from the Global Parkinson’s Genetics Program (GP2; https://gp2.org). Specifically, we used Tier 2 data from GP2 releases 6 [10.5281/zenodo.10472143] and 7 [10.5281/zenodo.10962119]. Tier 1 data can be accessed by completing a form on the Accelerating Medicines Partnership in Parkinson’s Disease (AMP®-PD) website (https://amp-pd.org/register-for-amp-pd). Tier 2 data access requires approval and a Data Use Agreement signed by your institution. All GP2 data is hosted in collaboration with the Accelerating Medicines Partnership in Parkinson’s disease, and is available via application on the website (https://amp-pd.org/register-for-amp-pd;10.5281/zenodo.7904832). Genotyping imputation, quality control, ancestry prediction, and processing was performed using GenoTools v1.0, publicly available on GitHub (https://github.com/GP2code/GenoTools).

## Code availability

All code generated for this article, and the identifiers for all software programs and packages used, are available on GitHub [https://github.com/GP2code/MAPThaplotype] and were given a persistent identifier via Zenodo [10.5281/zenodo.15933056].
