# Supplementary material for: Exploring MAPT-containing H1 and H2 haplotypes in Parkinson’s disease across diverse populations
Source: NPJ Parkinsons Dis. 2026 Jun 24;12:187. doi: 10.1038/s41531-026-01394-9 (PMC13452820; doi:10.1038/s41531-026-01394-9)
Supplement: Supplementary file 1 [file 41531_2026_1394_MOESM1_ESM.docx]

# Exploring MAPT-containing H1 and H2 haplotypes in Parkinson's Disease across diverse populations

## Supplementary Figures

**Supplementary Figure 1: Forest plot showing odds ratio (OR) estimates from the non-adjusted association analyses with a 95% confidence interval for *MAPT* H1 haplotype on Parkinson’s disease risk in the GP2 ancestry groups and LARGE-PD.** N shows the sample size per population. P-values are marked with stars, where one corresponds to p<0.1, and three stars show p<0.05. A dashed red line is marked at OR = 1.

**Supplementary figure 2**: **Forest plot showing odds ratio (OR) estimates from the association analyses with a 95% confidence interval for *MAPT* H1 haplotype on Parkinson’s disease risk in the GP2 ancestry groups and LARGE-PD, adjusting for age, sex and principal components 1-5.** N shows the sample size per population. P-values are marked with stars, where one corresponds to p<0.1, and three stars show p<0.05. A dashed red line is marked at OR = 1.

**A)**

**B)**

**Supplementary figure 3**: **Frequency of subhaplotypes in *MAPT,* comparing available whole genome sequencing (WGS) data and imputed genotype data in the EUR ancestry group for A) PD patients and B) controls.** Data was available for 452 PD patients and 92 controls. Only subhaplotypes with a minor allele frequency (MAF) >0.5% in the patients or controls are shown. The MAF are sorted based on frequency in the patient group.

**A)**

**B)**

**Supplementary figure 4**: **Frequency of subhaplotypes in *MAPT,* comparing whole genome sequencing (WGS) data and imputed genotype data in the AFR ancestry group for A) PD patients and B) controls.** Data was available for 99 PD patients and 51 controls. Only subhaplotypes with a minor allele frequency (MAF) >0.5% in the patients or controls are shown. The MAF are sorted based on frequency in the patient group.

**A)**

**B)**

**C)**

**D)**

**E)**

**F)**

**G)**

**H)****I)**

**Supplementary Figure 4. Regional plot of variant associations at the *MAPT* locus in each GP2 ancestry group**. Association results are derived from an adjusted regression model with a MAF filter of >1%. The y-axis represents –log₁₀(p) values, while the x-axis corresponds to genomic positions. The maximum y value is adjusted to the maximum –log₁₀(p) value derived from the dataset. Significant variants are highlighted in the *MAPT* region (chr17:45,794,527–46,228,334) with annotations of rs IDs, including the H2-tagging SNP (rs1052553) marked by a vertical gray dotted line. The genome-wide significance threshold is shown as a red dashed line.

## Supplementary Data

Supplementary Data are provided in a separate file but enlisted here.

Supplementary Data 1. Summary of clinical data / demographics of PD patients and controls in the study
Supplementary Data 2. Power calculations
Supplementary Data 3. Frequency of H1/H2 haplotypes across the populations in PD patients and controls
Supplementary Data 4. Hardy-Weinberg Equilibrium (HWE) p-values for rs1052553
Supplementary Data 5. Unadjusted association results between the H1 haplotype and PD in the differenct ancestry groups
Supplementary Data 6. Adjusted association results (age, sex, PC1-5) between the H1 haplotype and PD in the differenct ancestry groups
Supplementary Data 7. Adjusted association results (sex, PC1-5) between the H1 haplotype and PD in the differenct ancestry groups
Supplementary Data 8. Age and age at onset (AAO) for PD patients carrying the H1 vs H2 MAPT haplotype
Supplementary Data 9. Linear regression results for PD AAO
Supplementary Data 10. Hardy-Weinberg equilibrium (HWE) calculations for MAPT variants used for generating subhaplotypes
Supplementary Data 11. Frequency of H1/H2 subhaplotypes across populations in PD cases and controls
Supplementary Data 12. Subhaplotype ranking per ancestry group
